# The role of diet in diabetes gastroparesis treatment: a systematic review and meta-analysis

**DOI:** 10.3389/fendo.2024.1379398

**Published:** 2024-06-18

**Authors:** Dezhi Lin, Hui Wang, Yangxu Ou, Longlong Li, Qiang Zhang, Jiayin Yan, Dezhong Peng, Sihan Peng

**Affiliations:** ^1^ School of Acupuncture and Tuina, Chengdu University of Traditional Chinese Medicine, Chengdu, China; ^2^ Department of Rehabilitation, Zhongjiang County Hospital of Traditional Chinese Medicine, Deyang, China; ^3^ Department of Endocrinology, Hospital of Chengdu University of Traditional Chinese Medicine, Chengdu, China; ^4^ TCM Regulating Metabolic Diseases Key Laboratory of Sichuan Province, Hospital of Chengdu University of Traditional Chinese Medicine, Chengdu, China

**Keywords:** diabetes, gastroparesis, diet, systematic review, meta-analysis

## Abstract

**Background:**

Diabetic gastroparesis is a common complication in patient with diabetes. Dietary intervention has been widely used in the treatment of diabetic gastroparesis. The aim of this study is to evaluate the role of diet in the treatment of diabetic gastroparesis.

**Methods:**

This systematic review was conducted a comprehensive search of randomized controlled trials using dietary interventions for the treatment of diabetic gastroparesis up to 9 November 2023. The primary outcomes were gastric emptying time and clinical effect, while fasting blood glucose, 2-hour postprandial blood glucose and glycosylated hemoglobin were secondary outcomes. Data analysis was performed using RevMan 5.4 software, and publication bias test was performed using Stata 15.1 software.

**Results:**

A total of 15 randomized controlled trials involving 1106 participants were included in this review. The results showed that patients with diabetic gastroparesis benefit from dietary interventions (whether personalized dietary care alone or personalized dietary care+routine dietary care). Compared with routine dietary care, personalized dietary care and personalized dietary care+routine dietary care can shorten the gastric emptying time, improve clinical efficacy, and reduce the level of fasting blood glucose, 2-hour postprandial blood glucose and glycosylated hemoglobin.

**Conclusions:**

Limited evidence suggests that dietary intervention can promote gastric emptying and stabilize blood glucose control in patients with diabetic gastroparesis. Dietary intervention has unique potential in the treatment of diabetic gastroparesis, and more high-quality randomized controlled trials are needed to further validate our research results.

**Systematic review registration:**

https://www.crd.york.ac.uk/prospero/, identifier CRD42023481621.

## 1 Introduction

Diabetes gastroparesis (DGP) is a common complication of diabetes. The symptoms and objective evidence of DGP suggest food retention in the stomach, delayed gastric emptying without mechanical obstruction, and clinical manifestations mainly include postprandial satiety, nausea, vomiting and epigastric pain (1). According to the data of the International diabetes Federation (IDF), the prevalence rate of diabetes is 10.5%, showing an increasing trend year by year, by 2030, there will be 643 million adults with diabetes (2). The prevalence of DGP is positively related to the prevalence of diabetes, and DGP will become more and more common with the increase of diabetes patients (3). Research has shown that DGP patients have poor quality of life and blood sugar control (4), and long-term treatment is needed to effectively control symptoms (5). In addition, compared with diabetes patients without gastroparesis, the mortality of DGP patients will increase due to the occurrence of cardiovascular events (6). Therefore, DGP has become a serious social public health issue that affects the health and quality of life of patients.

At present, the treatment of DGP includes drug therapy, gastric electrical stimulation, endoscopic therapy, surgical treatment, and dietary therapy, all of which can improve gastric emptying (7). However, clinicians mainly focus on gastric prokinetic drugs for the treatment of DGP, such as domperidone, metoclopramide, and mosapride citrate (8, 9). Although these drugs can help alleviate gastrointestinal discomfort in DGP patients, long-term use of them may also cause adverse reactions such as tardade dyskinesia (1). Some DGP patients also choose gastric electrical stimulation therapy, which can improve gastric emptying but can lead to weight gain (10). In addition, endoscopic or surgical treatment is the last choice for DGP patients, used for some refractory DGP (11, 12), but the long-term effects are not satisfactory and increase the economic burden on patients and their families.

As a green, safe, simple and effective alternative therapy, dietary therapy plays an important role in preventing people with impaired fasting blood glucose or impaired glucose tolerance from developing type 2 diabetes (13, 14), and has been widely used in the treatment of DGP. Studies (15, 16) have shown that dietary adjustments are beneficial for DGP patients. The American College of Gastroenterology (ACG) guidelines for gastroparesis also recommend dietary management for DGP patients to correct their nutritional status, increase the likelihood of symptom relief, enhance gastric emptying, and improve their blood sugar control (1). However, patients receive dietary interventions based on physiological principles rather than clinical evidence (17). Therefore, it is particularly important to study the role of diet in DGP treatment based on clinical evidence. At present, it seems that no researchers have summarized and analyzed the clinical evidence of dietary intervention in DGP.

Therefore, it is necessary to improve the understanding of the available evidence by integrating and analyzing the existing clinical evidence of dietary therapy for DGP to help recommend the best dietary interventions for the treatment of DGP. This study included randomized controlled trials of using diet to intervene for patients with DGP and conducted a systematic review and meta-analysis of them, aiming to observe the changes in gastric emptying time (GET), clinical effect (CE), fasting blood glucose (FBG), 2-hour postprandial blood glucose (2hPBG), and glycosylated hemoglobin (HbA1c) levels in patients with DGP after dietary intervention, and to explore the effects of dietary intervention on gastric emptying and blood glucose control in patients with DGP.

## 2 Methods

### 2.1 Registration

**Figure 1:**
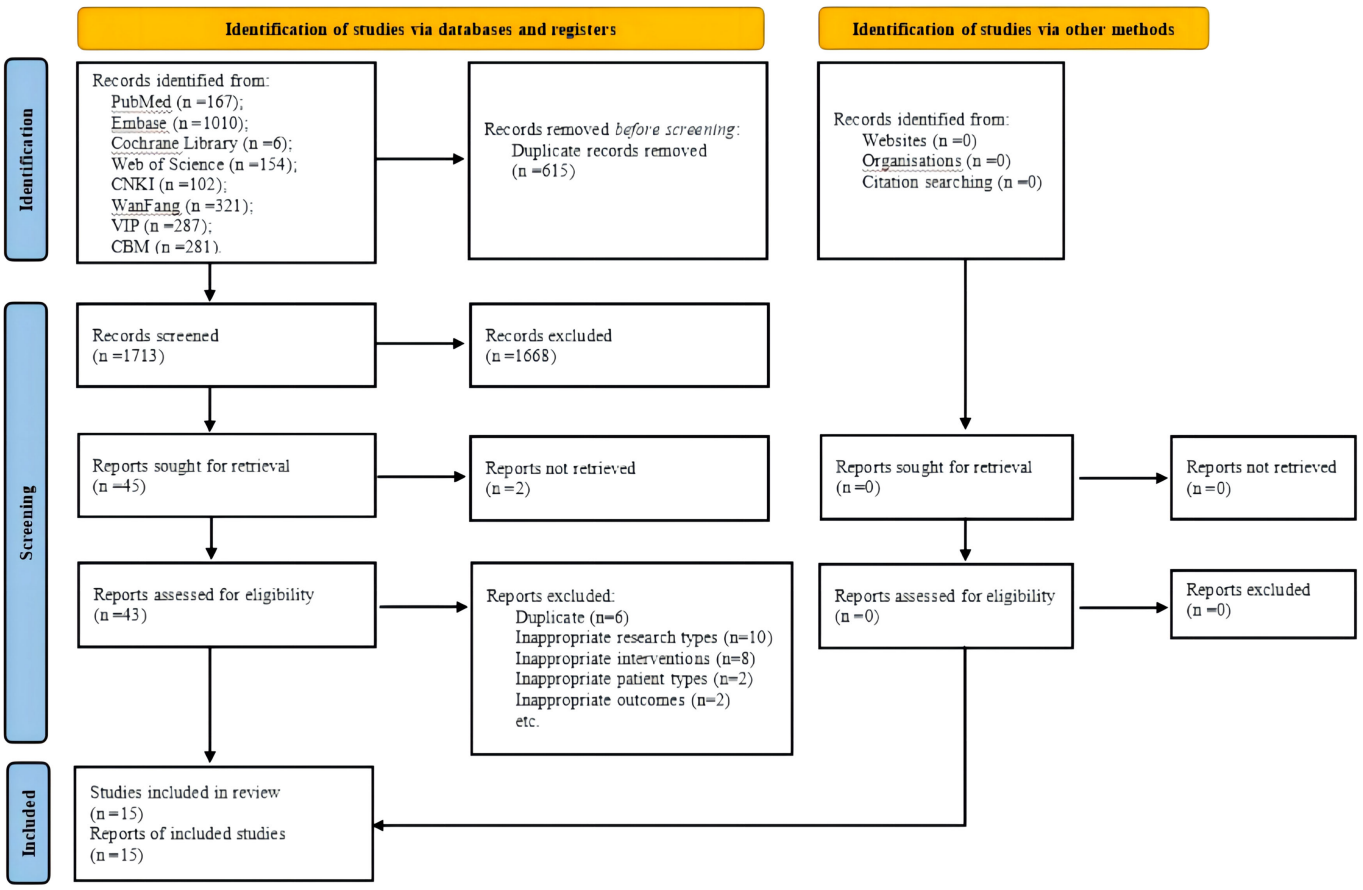
PRISMA flow chart. CNKI, China National Knowledge Infrastructure; VIP, Chongqing VIP Database; CBM, Chinese Biomedical Literature Database.

This review was performed in accordance with the Cochrane Handbook for Systematic Reviews of Interventions (18), and the format was followed the Preferred Reporting Items for Systematic reviews and Meta-analyses (19). We mentioned the reference to the PRISMA flow chart (**Figure 1**). The registered study protocol of this review was published in PROSPERO (registration number: CRD42023481621).

### 2.2 Search strategy

Two authors (HW and YO) systematically searched four English language databases (PubMed, Cochrane Library, EMBASE, and Cochrane Library, and Web of Science) and four Chinese databases (China National Knowledge Infrastructure, Chongqing VIP Database, Wanfang Database, and Chinese Biomedical Literature Database). The eight databases were searched for studies published from the time of database establishment until 9 November 2023. The search strategy combined the main keywords such as “diabetes mellitus”, “gastroparesis”, and “diet, food, and nutrition”. The strategy used to search PubMed databases is shown in **Table 1**, which has been modified to optimize the search of other databases. The authors also used the reference list of related articles and review articles to find cited articles that were not found in the database search.

**Table 1:** Search strategy for the PubMed database.

#1	“Diet, Food, and Nutrition”[MeSH Terms]
#2	(((((((((((((Diet[Title/Abstract]) OR (Nutrition[Title/Abstract])) OR (Food[Title/Abstract])) OR (Diet Therapy[Title/Abstract])) OR (Restrictive Diet Therapy[Title/Abstract])) OR (Restriction Diet Therapy[Title/Abstract])) OR (Dietary Restriction[Title/Abstract])) OR (Dietary Modification[Title/Abstract])) OR (Diet Modification[Title/Abstract])) OR (Diet Nursing[Title/Abstract])) OR (Dietary intervention[Title/Abstract])) OR (Nutrition Therapy[Title/Abstract])) OR (Nutritional Support[Title/Abstract])))
#3	#1 OR #2
#4	“Diabetes Mellitus”[MeSH Terms]
#5	(((((((Diabetes[Title/Abstract]) OR (Diabetes Insipidus[Title/Abstract])) OR (Diet, Diabetic[Title/Abstract])) OR (Prediabetic State[Title/Abstract])) OR (Scleredema Adultorum[Title/Abstract])) OR (Glycation End Products, Advanced[Title/Abstract])) OR (Glucose Intolerance[Title/Abstract]))))
#6	#4 OR #5
#7	“Gastroparesis”[MeSH Terms]
#8	((((((stomach paresis[Title/Abstract]) OR (Gastropareses[Title/Abstract])) OR (Gastric Stasis[Title/Abstract])) OR (Gastric Stases[Title/Abstract])) OR (Stases, Gastric[Title/Abstract])) OR (Stasis, Gastric[Title/Abstract])))
#9	#7 OR #8
#10	#3 AND #6 AND #9

### 2.3 Study selection

#### 2.3.1 Eligibility criteria

1. Patients with DGP over the age of 18 years (No restriction on the type of diabetes).
2. Intervention (s): Personalized dietary intervention, dietary patterns, or personalized dietary care (PDC) combined with routine dietary care (RDC).
3. Comparator(s)/control: All interventions, placebo or routine care.
4. Outcomes: Primary outcomes are GET and CE. Secondry outcomes included FBG, 2hPBG, and HbA1c.
5. Study design: Randomized controlled trials of using diet to intervene for patients with DGP.

#### 2.3.2 Exclusion criteria

1. Studies with repeated data or secondary analysis.
2. Study type does not match (including animal studies, master and doctoral dissertations, books, protocols, conference abstracts, case reports, correspondence, overview, or systematic review).
3. Non-DGP.
4. The therapy of the intervention group was non-personalized dietary intervention, dietary patterns, or personalized dietary intervention combined with routine dietary intervention.
5. Outcome indicators do not match.
6. Language is not Chinese or English.

### 2.4 Data extraction

Two investigators (LL, QZ and JY) independently extracted data from the included studies using a self-defined standardized extraction format. The extracted data included the name of the primary investigator, publication year, diabetes type, patient age, the country in which the study was performed, study design, dietary intervention mode, type of control intervention, the sample size of each group, intervention duration, randomization, allocation concealment and blinding methods, clinical variables, etc. Any disagreements were solved by consensus between the two investigators. In the event that a consensus could not be reached, a third investigator (DP) made the final judgment. When information was missing from a study, the corresponding author was contacted if the information necessary for correspondence was available.

### 2.5 Risk-of-bias assessment

Based on the RevMan 5.4 software built-in risk bias assessment tool provided by the Cochrane Collaboration (20), two researchers (YO and LL) evaluated the methodological quality of the included studies. The following seven aspects were chiefly included: 1) random sequence generation (selection bias); 2) allocation concealment (selection bias); 3) performance bias: blinded implementation (including participants, investigators, and outcome assessors); 4) detection bias: blinded evaluation of study results; 5) attrition bias: outcome data integrity; 6) reporting bias: selective reporting of results; 7) other biases. All the above biases were assessed and classified as low, unclear, or high risk of bias. Disagreements were discussed between the two reviewers, and if disagreements were not resolved between the two reviewers, a third reviewer (SP) participated in the discussion until a consensus was reached.

### 2.6 Evidence quality assessment of included studies

The Grading of Recommendations Assessment, Development, and Evaluations (GRADE) approach was used to rate the overall quality of evidence (21). The GRADE guideline provides evidence ratings for five aspects of included studies: risk of bias, inconsistency, indirectness, imprecision, and publication bias. The GRADE was divided into four levels of gradings for evidence quality: high, moderate, low, and very low. Two researchers (DL and HW) independently performed the assessment, and a third researcher (SP) then reviewed the evaluation. Any disagreement was resolved by discussion with a professional.

### 2.7 Data analysis

Statistical analysis was performed using RevMan (version 5.4). Continuous variables were evaluated using the mean difference (MD) with a 95% confidence interval (CI) when the measurement methods and units of the outcome indicators included in the study were the same, and the odds ratio (OR) values were used to evaluate the dichotomous variables. Evaluate the heterogeneity among included studies through I^2^. If I^2^ was ≤ 50% in the results, use a fixed-effects model. If I^2^ was > 50% in the results, there is significant heterogeneity between a group of studies, a random-effects model should be adopted to pool the data, and the sources of heterogeneity should be explored. Sensitivity analysis was performed by excluding each RCT sequentially from a group of studies, and comparing the model characteristics to test the robustness of the result. A funnel plot was used to evaluate the publication bias if more than nine trials were included in the meta-analysis. The asymmetry of the funnel plots was evaluated using Egger’s tests, and a P-value of < 0.05 represented significant publication bias (22).

## 3 Results

### 3.1 Search results

A total of 2328 potentially eligible articles were identified. After removing 615 duplicates and screening the 1713 remaining articles, 43 candidate studies were isolated. Review of the full text of each shortlisted study based on the eligibility criteria excluded 28 studies. Eventually, 15 studies (23–37) were included in this meta-analysis (**Figure 1**).

### 3.2 Study characteristics

The main characteristics of the 15 included studies are summarized in **Table 2**.

**Table 2:** Detailed characteristics of included trials.

Study	Study design	Type of diabetes	Duration of diabetes (years) (I/C)	Number (I/C)	Age (Years) (I/C)	Gender(M/F)		Clinical variables
						I	C	
An FL, 2021 (23)	RCT	type 2 diabetes	NR/NR	26/26	65.28±2.21/65.31±2.15	13/13	14/12	CE
Bao MM, 2016 (24)	RCT	type 2 diabetes	10.5±2.6/10.5±2.6	40/40	48.0±14.4/48.0±14.4	25/55		GET
Hu GF et al., 2022 (25)	RCT	NR	5.15±1.06/4.94±1.17	45/45	51.39±4.77/52.17±4.06	26/19	18/27	GET
Ji CY, 2018 (26)	RCT	NR	NR/NR	25/25	60.0±5.5/60.5±5.6	15/10	14/11	FBG, GET, CE
Liu ZY, 2021 (27)	RCT	NR	7.65±3.01/7.44±3.11	41/41	64.6±13.2/62.37±14.16	20/21	22/19	FBG, 2hPBG, HbA1c, GET
Li W, 2023 (28)	RCT	NR	NR/NR	50/50	52.69±5.45/52.52±5.11	27/23	22/28	FBG, 2hPBG, HbA1c, GET, CE
Pan ML et al., 2014 (29)	RCT	NR	5.1±1.6/5.4±1.7	40/40	70.2±6.1/69.8±6.9	23/17	21/19	GET, CE
Sun XL, 2015 (30)	RCT	NR	NR/NR	24/24	65.4±5.1/67.7±5.5	13/11	14/10	GET, CE
Wang CX et al., 2017 (31)	RCT	type 2 diabetes	7.1±1.8/6.9±1.6	36/36	67.1±5.1/66.8±4.9	21/15	19/17	CE
Wan Q et al., 2014 (32)	RCT	NR	4-19/5-16	20/20	45.93±10.32/46.73±10.85	10/10	8/12	FBG, 2hPBG, HbA1c, CE
Xu J, 2016 (33)	RCT	NR	4.5±2.9/4.6±2.3	48/48	57.2±3.9/56.3±4.2	27/21	26/22	FBG, 2hPBG, GET, CE
Xu J, 2018 (34)	RCT	NR	9.6±3.3/9.7±3.1	30/30	46.13±10.57/45.79±10.63	12/18	13/17	FBG, 2hPBG, HbA1c, GET
Xu LY et al., 2017 (35)	RCT	NR	10.6±1.2/10.6±1.2	38/38	69.2±5.0/69.2±5.0	42/34		CE
Yu CJ et al., 2022 (36)	RCT	type 2 diabetes	8.76±2.07/8.35±2.22	40/40	50.18±9.66/50.90±9.51	17/23	19/21	FBG, 2hPBG
Zhang Q et al., 2023 (37)	RCT	type 2 diabetes	8.49±2.08/8.92±2.06	50/50	50.64±9.06/50.21±9.05	22/28	24/26	FBG, 2hPBG

I, intervention group; C, control group; M, male; F, female; RCT, randomized controlled trial; NR, not reported; FBG, fasting blood glucose; 2hPBG, 2-hour postprandial blood glucose; HbA1c, glycosylated hemoglobin; GET, gastric emptying time; CE, clinical effect.

**Patient characteristics:** The 15 RCTs (23–37) included 1106 patients diagnosed with DGP, 547 of whom were male and 559 of whom were female. In terms of type of diabetes, five studies (23, 24, 31, 36, 37) included patients with type 2 diabetes, and the other 10 studies did not mention the classification of diabetes. In terms of geographical distribution, all participants are from China.

**Intervention characteristics:** The intervention group involves two dietary related therapies, namely PDC (25, 28, 29, 32, 34) and PDC combined with RDC (23, 24, 26, 27, 30, 31, 33, 35–37). Although there are differences between each study on PDC, there are the following consensus: 1) Retrospective dietary investigation was conducted to evaluate the nutritional status of DGP patients, and to educate them on diet and nutrient-related health knowledge; 2) Develop personalized recipes based on the patient’s physical condition; 3) Adjust food composition: advocate a low-fiber diet; 4) Adjust food form: the diet is mainly semi-liquid or liquid foods, and avoid the intake of non-digestible foods such as frying and high fat; 5) Adjust the number of meals: follow the principle of “a little each time but many times”; 6) Adjust the meal time and insulin injection time based on the patient’s postprandial blood glucose changes and gastric emptying. The specific intervention details of the intervention group are shown in **Table 3**.

**Table 3:** The grouping of the included studies and the specific intervention details of each group.

Study	Intervention group	Control group	Treatment duration	Specific intervention details (I)	Specific intervention details (C)
An FL, 2021 (23)	PDC+RDC	RDC	NR	1.Food form adjustment, guide patients to take in low fiber, liquid, and semi liquid foods. 2.Food composition adjustment, control the intake of high fiber foods, reduce the intake of fried, solid or high-fat foods, and adjust the diet according to different complications of patients. 3.Adjust the number of meals, follow the principle of “a little each time but many times”, and adjust the number of meals according to the patient's recovery situation.	1.Provide health education, inform patients of the causes of the disease and subsequent treatment measures, and enhance their health awareness. 2.Evaluate the patient's psychological state, provide psychological care to the patient, and keep them optimistic.
Bao MM, 2016 (24)	PDC+RDC	RDC	2w	1.Retrospective dietary investigation was conducted to evaluate the nutritional status of patients. 2.Develop personalized recipes. 3.Ensure balanced nutrition. 4.Coordinate insulin injection time and eating time, the more severe the gastroparesis symptoms, the longer the interval between insulin injection time and meal time. 5.Health education.	1.Use insulin pump or intravenous insulin injection to control blood sugar, take mosapride citrate tablets orally. 2.Guide patients to consume liquid or semi liquid foods. 3.Reduce or prohibit the consumption of high-fiber vegetables, high-acid foods and gas-producing foods. 4.Avoid consuming stimulating foods. 5.Follow the principle of “a little each time but many times”. 6.Strictly monitor blood sugar and observe whether hypoglycemia occurs.
Hu GF et al., 2022 (25)	PDC	RDC	8w	Implementing dietary care based on the PDCA cycle: 1.Plan phase (Plan). Discover and evaluate patient information and analyze health status. Establish a plan for the etiology: ①Help patients establish correct cognitive concepts about DGP through health education; ②Guide patients to participate in all activities; ③Through educational activities or manuals, help patients understand the correct and healthy measurement methods for DGP diet, and popularize the range of DGP diet; ④Distribute monitoring diaries to guide patients in recording monitoring indicators. 2.Execution phase (Do). ①Participate in DGP health education and provide personalized guidance to correct patient cognitive misunderstandings; ②Organize collective activities to enable patients to master DGP self-management skills. 3.Check phase (Check). Follow up to understand the improvement of the patient's dietary condition and inquire about their compliance. 4.Action phase (Action). Patients were followed up and feedback was analyzed to summarize and improve the intervention plans: ①Give affirmation to patients who strictly follow the plan, and strengthen specific measures; ②For patients who are difficult to strictly adhere to their dietary plan, the intervention plan can be gradually and reasonably modified as the next cycle goal, which can be implemented again to start the next PDCA cycle.	1.Advocate a low fiber diet. 2.The diet is mainly semi-liquid or liquid foods. 3.Follow the principle of “a little each time but many times”, and avoid the intake of non-digestible foods such as frying and high fat.
Ji CY, 2018 (26)	PDC+RDC	RDC	4w	1.Closely observe changes in the patient's vital signs. 2.Develop personalized recipes based on the patient's physical condition. 3.The diet should be balanced and follow the principle of “a little each time but many times”. 4.Guide patients to consume liquid or semi liquid foods. 5.Adjust the meal time and insulin injection time based on the patient's blood glucose change curve every 30 minutes after meals and the gastric emptying rate detected by ultrasound. If the gastric emptying rate is less than 60%, eat within 15 minutes after insulin injection.	1.Provide daily medication, monitor changes in blood sugar and vital signs. 2.Conduct routine education and guidance on DGP related knowledge, diet and exercise.
Liu ZY, 2021 (27)	PDC+RDC	RDC	12w	1.The research team formulated and revised the daily diagnosis and treatment plan, and guided patients to master the application of personalized recipes. 2.Investigate the patient's dietary habits and correct any unhealthy dietary habits. 3.Distribute dietary record sheets to record daily dietary content. 4.According to the Chinese Guidelines for Medical Nutrition Treatment of diabetes to develop an personalized diet plan, and control the total intake of the three major nutrient elements (carbohydrates, fats, proteins).	1.Apply insulin treatment. 2.Oral administration of mosapride citrate tablets. 3.Combine with aerobic exercise. 4.Master DGP related knowledge through health education and change unhealthy lifestyles. 5.Calculate dietary intake based on standard body mass, follow the principle of “a little each time but many times” and regular meals, and try to avoid high cholesterol foods as much as possible.
Li W, 2023 (28)	PDC	RDC	8w	Implementing dietary care based on the PDCA cycle: 1.Plan phase (Plan). Discover and evaluate patient information and analyze health status. Establish a plan for the etiology: ①Help patients establish correct cognitive concepts about DGP through health education; ②Guide patients to participate in all activities; ③Through educational activities or manuals, help patients understand the correct and healthy measurement methods for DGP diet, and popularize the range of DGP diet; ④Distribute monitoring diaries to guide patients in recording monitoring indicators. 2.Execution phase (Do). ①Participate in DGP health education and provide personalized guidance to correct patient cognitive misunderstandings; ②Organize collective activities to enable patients to master DGP self-management skills. 3.Check phase (Check). Follow up to understand the improvement of the patient's dietary condition and inquire about their compliance. 4.Action phase (Action). Patients were followed up and feedback was analyzed to summarize and improve the intervention plans: ①Give affirmation to patients who strictly follow the plan, and strengthen specific measures; ②For patients who are difficult to strictly adhere to their dietary plan, the intervention plan can be gradually and reasonably modified as the next cycle goal, which can be implemented again to start the next PDCA cycle.	1.Advocate a low fiber diet. 2.The diet is mainly semi-liquid or liquid foods. 3.Follow the principle of “a little each time but many times”, and avoid the intake of non-digestible foods such as frying and high fat.
Pan ML et al., 2014 (29)	PDC	RDC	8w	1.Food composition adjustment, advocate a low fiber diet. 2.Food form adjustment, the diet is mainly semi-liquid or liquid foods, and avoid the intake of non-digestible foods such as frying and high fat. 3.Adjust the number of meals, follow the principle of “a little each time but many times”.	1.Guide patients to exercise reasonably and control their diet. 2.Control blood sugar with medication.
Sun XL, 2015 (30)	PDC+RDC	RDC	8w	1.Food composition adjustment, advocate a low fiber diet. 2.Food form adjustment, the diet is mainly semi-liquid or liquid foods, and avoid the intake of non-digestible foods such as frying and high fat. 3.Adjust the number of meals, follow the principle of “a little each time but many times”.	1.Provide health education and psychological care. 2.Guide patients to exercise reasonably and control their diet. 3.Strictly monitor the patient's blood sugar.
Wang CX et al., 2017 (31)	PDC+RDC	RDC	12w	1.Adjust the number of meals, follow the principle of “a little each time but many times”. 2.Food form adjustment, the diet is mainly semi-liquid or liquid foods. 3.Food composition adjustment, advocate a low fiber diet, and avoid the intake of non-digestible foods such as frying, high fiber and high fat. 4.Meal time adjustment, adjust the meal time and insulin injection time based on the patient's blood glucose change curve every 30 minutes after meals and the gastric emptying rate detected by ultrasound. If the gastric emptying rate is less than 60%, eat within 15 minutes after insulin injection; If the gastric emptying rate is between 60% and 70%, eat within 20 minutes after insulin injection. 5.Strengthen nutritional support: strengthen the propaganda and education of nutrition knowledge, and add enough nutrition on the basis of strict control of blood sugar.	1.Control blood sugar with medication and engage in moderate exercise. 2.Calculate dietary intake based on standard body mass, follow the principle of “a little each time but many times” and regular meals, and try to avoid high cholesterol foods as much as possible.
Wan Q et al., 2014 (32)	PDC	RDC	12w	1.Apply insulin treatment. 2.Oral administration of mosapride citrate tablets. 3.Psychological support. 4.A retrospective dietary survey of patients was conducted by a nutritionist to understand the patient's dietary habits, frequency of eating, and provide health education. 5.Develop personalized recipes. 6.The principle of recipe formulation: ①Control the total intake of the three major nutrient elements (carbohydrates, fats, proteins); ②Follow the principle of “a little each time but many times”; ③The patient's food intake was proportional to the amount of exercise; ④Reduce the intake of indigestible vegetables; ⑤Prohibit the consumption of stimulating foods; ⑥Guide patients and their families to massage the abdomen in a clockwise direction to help with gastrointestinal peristalsis and promote digestion; ⑦Strictly monitor blood sugar and observe whether hypoglycemia occurs.	1.Apply insulin treatment. 2.Oral administration of mosapride citrate tablets. 3.Implement diet guidance according to diabetes nursing routine, and supervise their diet implementation.
Xu J, 2016 (33)	PDC+RDC	RDC	8w	1.Retrospective dietary investigation was conducted to assess the nutritional status of patients and calculate the daily dietary requirements. 2.Follow the principle of “a little each time but many times”. 3.Adjust the meal time and insulin injection time based on the patient's blood glucose change curve every 30 minutes after meals and the gastric emptying rate detected by ultrasound. If the gastric emptying rate is less than 60%, eat within 15 minutes after insulin injection; If the gastric emptying rate is between 60% and 70%, eat within 20 minutes after insulin injection. 4.Guide patients to consume liquid or semi liquid foods. 5.Reduce the content of fiber in food. 6.Avoid fried and indigestible foods.	1.Actively control the patient's blood sugar level, provide health education, instruct them to develop good lifestyle habits. 2.Provide dietary adjustments. 3.Guide patients to engage in moderate exercise. 4.Provide psychological care to patients to maintain an optimistic attitude. 5.Regularly monitor the patient's blood sugar and control it through medication in a reasonable manner.
Xu J, 2018 (34)	PDC	RDC	NR	1.Apply conventional hypoglycemic drugs. 2.Psychological support. 3.A retrospective dietary survey of patients was conducted by a nutritionist to understand the patient's dietary habits, frequency of eating. 4.Develop personalized recipes. 5.The principle of recipe formulation: ①Control the total intake of the three major nutrient elements (carbohydrates, fats, proteins); ②Follow the principle of “a little each time but many times”; ③The patient's food intake was proportional to the amount of exercise; ④Reduce the intake of indigestible vegetables; ⑤Prohibit the consumption of stimulating foods; ⑥Guide patients and their families to massage the abdomen in a clockwise direction to help with gastrointestinal peristalsis and promote digestion; ⑦Strictly monitor blood sugar and observe whether hypoglycemia occurs. 6.Health education.	1.Apply conventional hypoglycemic drugs. 2. Adopt traditional dietary management.
Xu LY et al., 2017 (35)	PDC+RDC	RDC	12w	Adjust the meal time and insulin injection time based on the patient's blood glucose change curve every 30 minutes after meals and the gastric emptying rate detected by ultrasound. If the gastric emptying rate is less than 60%, eat within 15 minutes after insulin injection; If the gastric emptying rate is between 60% and 70%, eat within 20 minutes after insulin injection.	1.Exercise and insulin injection to control blood sugar. 2.Follow the principle of “a little each time but many times”. 3.Guide patients to consume liquid or semi liquid foods. 4. Eat a low fiber diet. 5. Avoid fried and indigestible foods.
Yu CJ et al., 2022 (36)	PDC+RDC	RDC	8w	1.A retrospective dietary survey of patients was conducted by a nutritionist to understand the patient's dietary habits, frequency of eating, and provide health education. 2.Develop personalized recipes. 3.The principle of recipe formulation: ①Control the total intake of the three major nutrient elements (carbohydrates, fats, proteins); ②Follow the principle of “a little each time but many times”; ③The patient's food intake was proportional to the amount of exercise; ④Reduce the intake of indigestible vegetables; ⑤Prohibit the consumption of stimulating foods; ⑥Guide patients and their families to massage the abdomen in a clockwise direction to help with gastrointestinal peristalsis and promote digestion; ⑦Strictly monitor blood sugar and observe whether hypoglycemia occurs.	1.Injecting insulin or taking oral hypoglycemic drugs. 2.Oral administration of mosapride citrate tablets. 3.Provide necessary psychological guidance. 4.Strictly adhere to a low salt, low fat, and low sugar diet. 5.Increase intake of fresh vegetables and reduce intake of saturated fatty acids and cholesterol. 6.Meal on time. 7.Guide the patient's exercise based on their physical condition.
Zhang Q et al., 2023 (37)	PDC+RDC	RDC	8w	1.A retrospective dietary survey of patients was conducted by a nutritionist to understand the patient's dietary habits, frequency of eating, and provide health education. 2.Develop personalized recipes. 3.The principle of recipe formulation: ①Control the total intake of the three major nutrient elements (carbohydrates, fats, proteins); ②Follow the principle of “a little each time but many times”; ③The patient's food intake was proportional to the amount of exercise; ④Reduce the intake of indigestible vegetables; ⑤Prohibit the consumption of stimulating foods; ⑥Guide patients and their families to massage the abdomen in a clockwise direction to help with gastrointestinal peristalsis and promote digestion; ⑦Strictly monitor blood sugar and observe whether hypoglycemia occurs.	1.Injecting insulin or taking oral hypoglycemic drugs. 2.Strictly adhere to a low salt, low fat, and low sugar diet. 3.Increase intake of fresh vegetables and reduce intake of saturated fatty acids and cholesterol. 4.Meal on time. 5.Guide the patient's exercise based on their physical condition.

I, intervention group; C, control group; PDC, personalized dietary care; RDC, routine dietary care; W, week; NR, not reported; DGP, diabetic gastroparesis.

**Control characteristics:** The control group only involved RDC. RDC mainly includes: 1) Injecting insulin or taking oral hypoglycemic drugs to control blood sugar levels; 2) Oral administration of mosapride citrate tablets; 3) Conduct routine education and guidance on DGP related knowledge, diet and exercise; 4) Provide psychological care to patients to maintain an optimistic attitude. The specific intervention details of the control group are shown in **Table 3**.

**Outcomes:** The role of diet in the treatment of DGP was evaluated by subjective or objective outcome indicators such as GET, CE, FBG, 2hPBG and HbA1c.

### 3.3 Risk of bias

**Figure 2:**
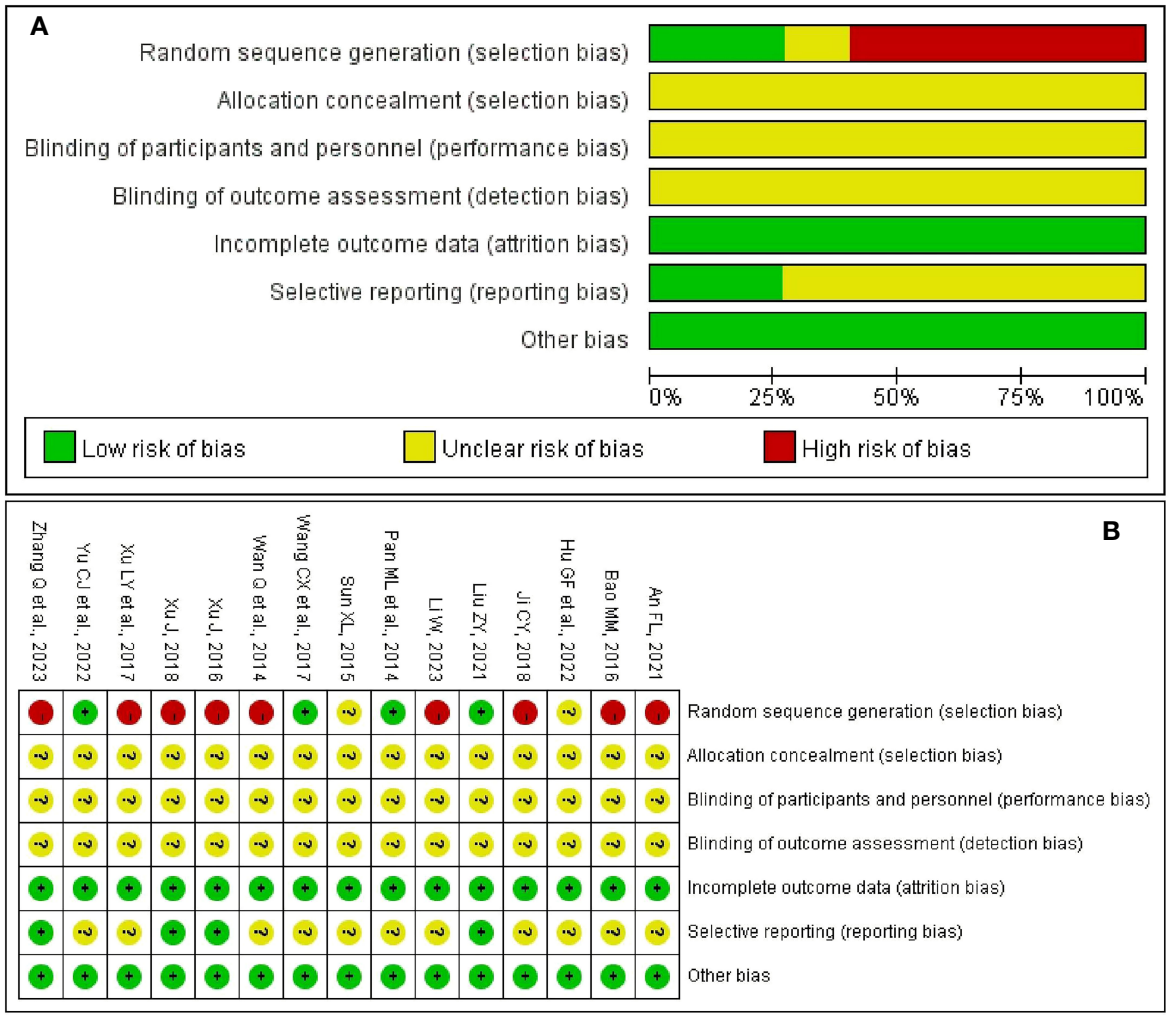
Risk of bias. **(A)** Risk of bias graph of included trials. **(B)** Risk of bias summary of included trials.

**Figure 2A** summarizes the risk of bias in the included studies from seven domains. In terms of random sequence generation, four studies (27, 29, 31, 36) explicitly reported low-risk randomization methods. None of the studies mentioned allocation concealment and blinding (including blinding to participants, researchers, and outcome assessors). All included RCTs reported complete outcome measures were rated as low risk. In the field of selective reporting, four trials (27, 33, 34, 37) were evaluated as having a low risk of bias. Regarding the other bias, none of the included studies had any other risk of bias. **Figure 2B** shows the risk of bias for each included trials.

### 3.4 Safety

Only one study (24) reported adverse events of diarrhea and constipation in the dietary intervention group, it may be related to the individual physical condition of the participants. No adverse events were reported in the remaining 14 trials (23, 25–37), it may be related to the fact that most of the included studies closely monitor blood sugar to prevent the occurrence of hypoglycemia.

### 3.5 Meta-analysis

#### 3.5.1 PDC vs. RDC

**Figure 3:**
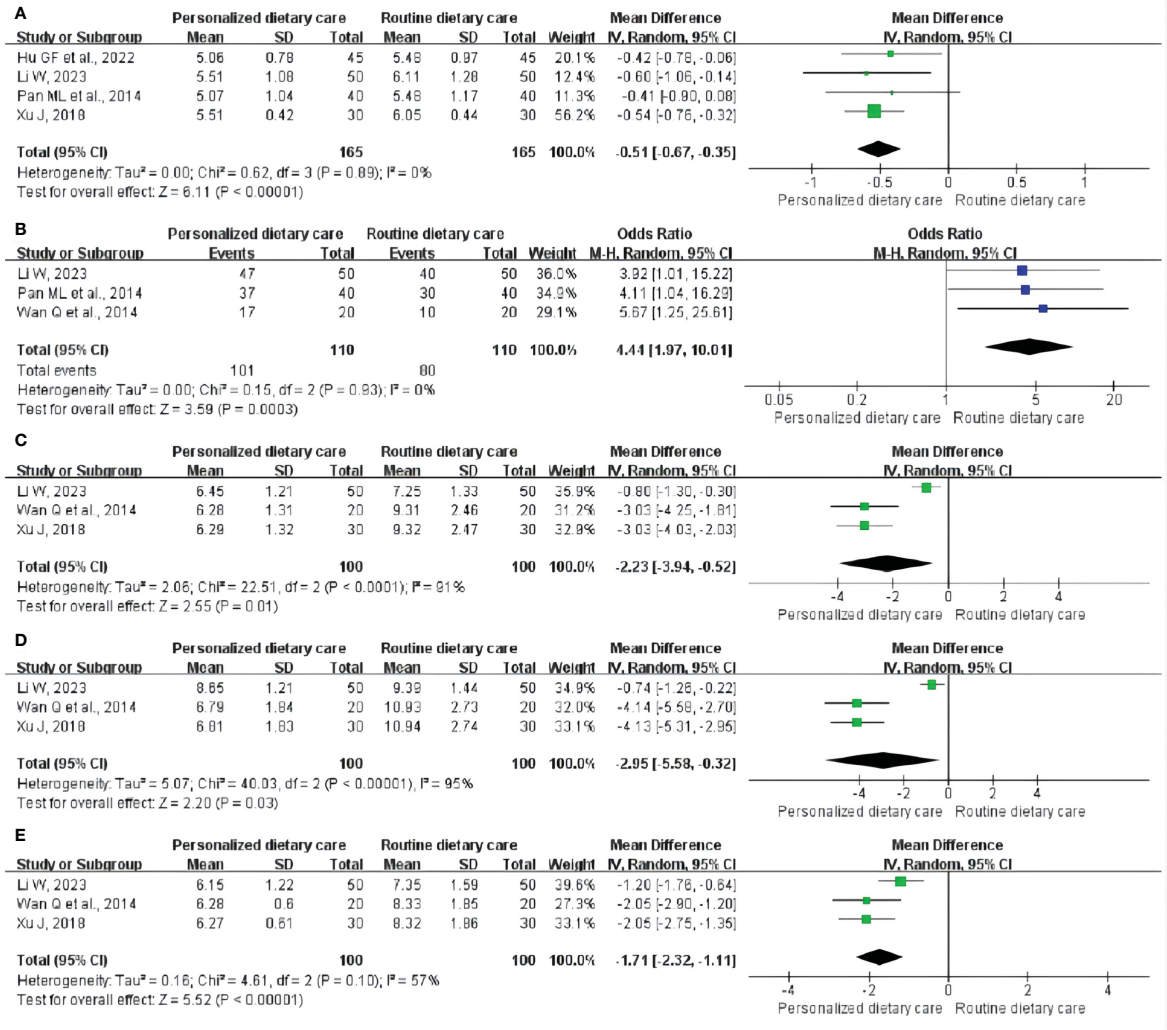
Meta-analysis of PDC versus RDC. **(A)** Meta-analysis of PDC versus RDC for the GET. **(B)** Meta-analysis of PDC versus RDC for the CE. **(C)**Metaanalysis of PDC versus RDC for the FBG. **(D)** Meta-analysis of PDC versus RDC for the 2hPBG. **(E)** Meta-analysis of PDC versus RDC for the HbA1c. PDC, personalized dietary care; RDC, routine dietary care; GET, gastric emptying time; CE, clinical effect; FBG, fasting blood glucose; 2hPBG, 2-hour postprandial blood glucose; HbA1c, glycosylated hemoglobin.

**GET:** Four studies (25, 28, 29, 34) included 330 participants and reported the changes in GET. Comprehensive analysis showed that PDC better shortened GET compared to RDC. (MD = -0.51; 95% CI: -0.67 to -0.35; P < 0.00001; I^2^ = 0%; **Figure 3A**).

**CE:** CE was observed in three trials (28, 29, 32) involving 220 participants. The analysis showed that compared with RDC, PDC could significantly improve CE in DGP treatment. (MD = 4.44; 95% CI: 1.97 to 10.01; P = 0.0003; I^2^ = 0%; **Figure 3B**).

**FBG:** Three RCTs (28, 32, 34) including 200 participants reported the results, showing that PDC reduced FBG levels in DGP patients compared with RDC. (MD = -2.23; 95% CI: -3.94 to -0.52; P = 0.01; I^2^ = 91%; **Figure 3C**).

**2hPBG:** Three studies (28, 32, 34) involving 200 participants reported 2hPBG. Compared with RDC, PDC could reduce the 2hPBG level in patients with DGP. (MD = -2.95; 95% CI: -5.58 to -0.32; P = 0.03; I^2^ = 95%; **Figure 3D**).

**HbA1c:** Three trials (28, 32, 34) included 200 participants and measured HbA1c levels. Compared to RDC, PDC reduced HbA1c levels in patients with DGP. (MD = -1.71; 95% CI: -2.32 to -1.11; P < 0.00001; I^2^ = 57%; **Figure 3E**).

#### 3.5.2 PDC+RDC vs. RDC

**Figure 4:**
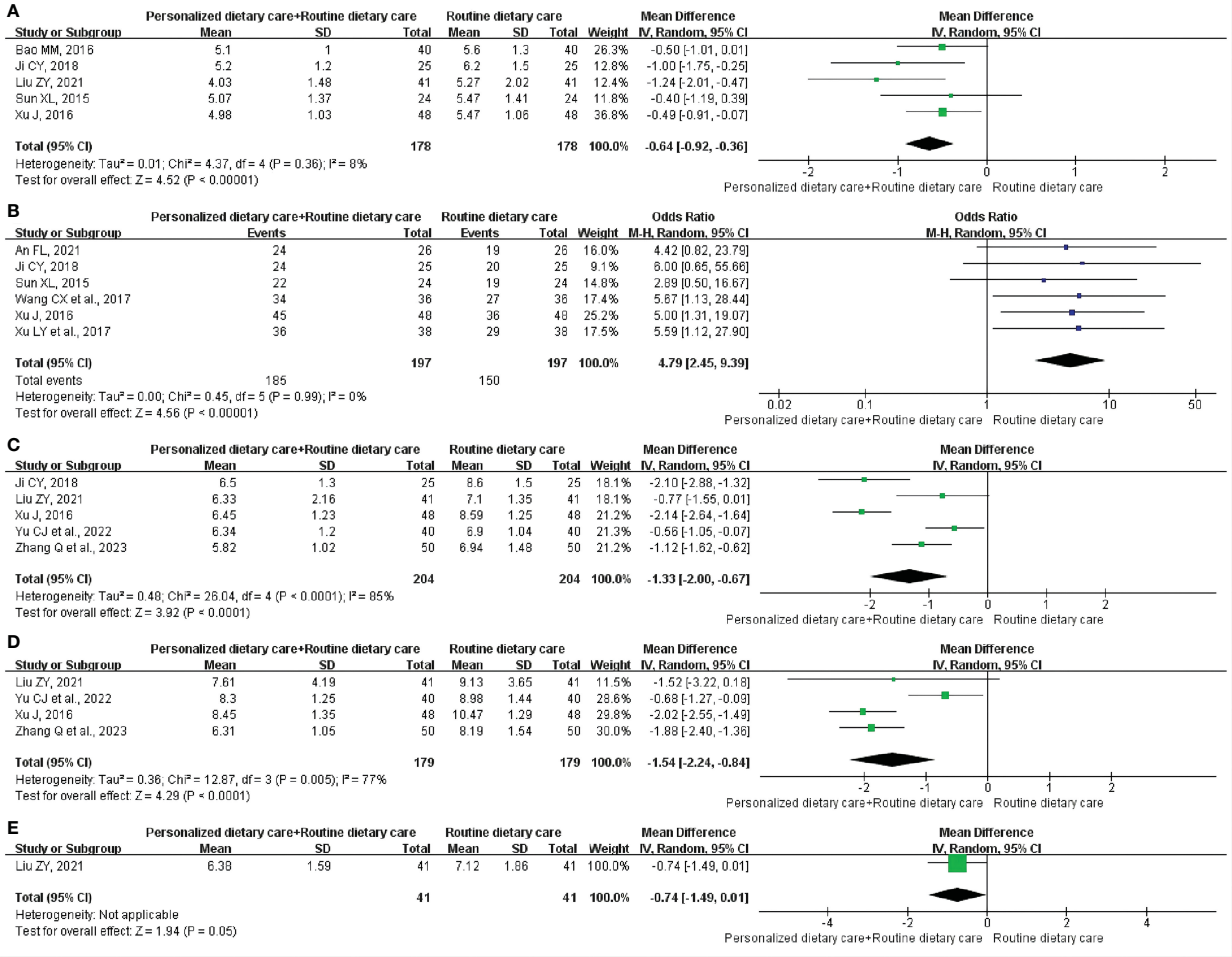
Meta-analysis of PDC+RDC versus RDC. **(A)** Meta-analysis of PDC+RDC versus RDC for the GET.**(B)** Meta-analysis of PDC+RDC versus RDC for the CE. **(C)** Metaanalysis of PDC+RDC versus RDC for the FBG. **(D)** Meta-analysis of PDC+RDC versus RDC for the 2hPBG. **(E)** Meta-analysis of PDC+RDC versus RDC for the HbA1c. PDC, personalized dietary care; RDC, routine dietary care; GET, gastric emptying time; CE, clinical effect; FBG, fasting blood glucose; 2hPBG, 2-hour postprandial blood glucose; HbA1c, glycosylated hemoglobin.

**GET:** Five studies (24, 26, 27, 30, 33) included 356 participants and reported the changes in GET. Comprehensive analysis showed that PDC+RDC better shortened GET compared to RDC. (MD = -0.64; 95% CI: -0.92 to -0.36; P < 0.00001; I^2^ = 8%; **Figure 4A**).

**CE:** CE was observed in six trials (23, 26, 30, 31, 33, 35) involving 394 participants. The analysis showed that compared with RDC, PDC+RDC could significantly improve CE in DGP treatment. (MD = 4.79; 95% CI: 2.45 to 9.39; P < 0.00001; I^2^ = 0%; **Figure 4B**).

**FBG:** Five RCTs (26, 27, 33, 36, 37) including 408 participants reported the results, showing that PDC+RDC reduced FBG levels in DGP patients compared with RDC. (MD = -1.33; 95% CI: -2.00 to -0.67; P < 0.0001; I^2^ = 85%; **Figure 4C**).

**2hPBG:** Four studies (27, 33, 36, 37) involving 358 participants reported 2hPBG. Compared with RDC, PDC+RDC could reduce the 2hPBG level in patients with DGP. (MD = -1.54; 95% CI: -2.24 to -0.84; P < 0.0001; I^2^ = 77%; **Figure 4D**).

**HbA1c:** One trial (27) included 82 participants and measured HbA1c levels. Compared to RDC, PDC+RDC reduced HbA1c levels in patients with DGP. (MD = -0.74; 95% CI: -1.49 to 0.01; P = 0.05; **Figure 4E**).

### 3.6 Sensitivity analysis

In the meta-analysis, we found that individual variables in the PDC and PDC+RDC groups had high heterogeneity, so we only conducted sensitivity analysis on these variables with high heterogeneity to explore the possible sources of heterogeneity. In the meta-analysis of PDC vs. RDC, we observed significant heterogeneity by omitting individual studies in variables such as FBG, 2hPBG, and HbA1c one by one. After excluding the study of Li W (28), the heterogeneity of research results related to FBG, 2hPBG, and HbA1c was significantly reduced (I^2 ^= 0%). In the meta-analysis of PDC+RDC vs. RDC, by omitting individual trials in the FBG variables one by one, we did not observe significant changes in the results, and these heterogeneity did not affect the stability of the results. When omitting individual RCTs in the 2hPBG variables one by one, we found that after excluding the study by Yu CJ et al. (36), heterogeneity was significantly reduced (I^2 ^= 0%). The specific details of sensitivity analysis are shown in **Table 4**.

**Table 4:** Sensitivity analysis.

Comparison	Outcome	Study omitted	Effect Estimate	Test for overall effect P-value	Heterogeneity I^2^ (%)
PDC VS. RDC	FBG	Li W, 2023 (28)	MD -0.80 [-1.30, -0.30]	<0.00001	0
		Wan Q et al., 2014 (32)	MD -3.03 [-4.25, -1.81]	=0.09	93
		Xu J, 2018 (34)	MD -3.03 [-4.03, -2.03]	=0.10	91
	2hPBG	Li W, 2023 (28)	MD -0.74 [-1.26, -0.22]	<0.00001	0
		Wan Q et al., 2014 (32)	MD -4.14 [-5.58, -2.70]	=0.16	96
		Xu J, 2018 (34)	MD -4.13 [-5.31, -2.95]	=0.16	95
	HbA1c	Li W, 2023 (28)	MD -1.20 [-1.76, -0.64]	<0.00001	0
		Wan Q et al., 2014 (32)	MD -2.05 [-2.90, -1.20]	=0.0002	71
		Xu J, 2018 (34)	MD -2.05 [-2.75, -1.35]	=0.0002	63
PDC+RDC VS. RDC	FBG	Ji CY, 2018 (26)	MD -2.10 [-2.88, -1.32]	=0.002	86
		Liu ZY, 2021 (27)	MD -0.77 [-1.55, 0.01]	=0.0002	88
		Xu J, 2016 (33)	MD -2.14 [-2.64, -1.64]	=0.0003	73
		Yu CJ et al., 2022 (36)	MD -0.56 [-1.05, -0.07]	<0.00001	79
		Zhang Q et al., 2023 (37)	MD -1.12 [-1.62, -0.62]	=0.002	88
	2hPBG	Liu ZY, 2021 (27)	MD -1.52 [-3.22, 0.18]	=0.0002	84
		Xu J, 2016 (33)	MD -2.02 [-2.55, -1.49]	=0.006	78
		Yu CJ et al., 2022 (36)	MD -0.68 [-1.27, -0.09]	<0.00001	0
		Zhang Q et al., 2023 (37)	MD -1.88 [-2.40, -1.36]	=0.009	82

MD, mean difference; PDC, personalized dietary care; RDC, routine dietary care; FBG, fasting blood glucose; 2hPBG, 2-hour postprandial blood glucose; HbA1c, glycosylated hemoglobin.

### 3.7 Publication bias

**Figure 5:**
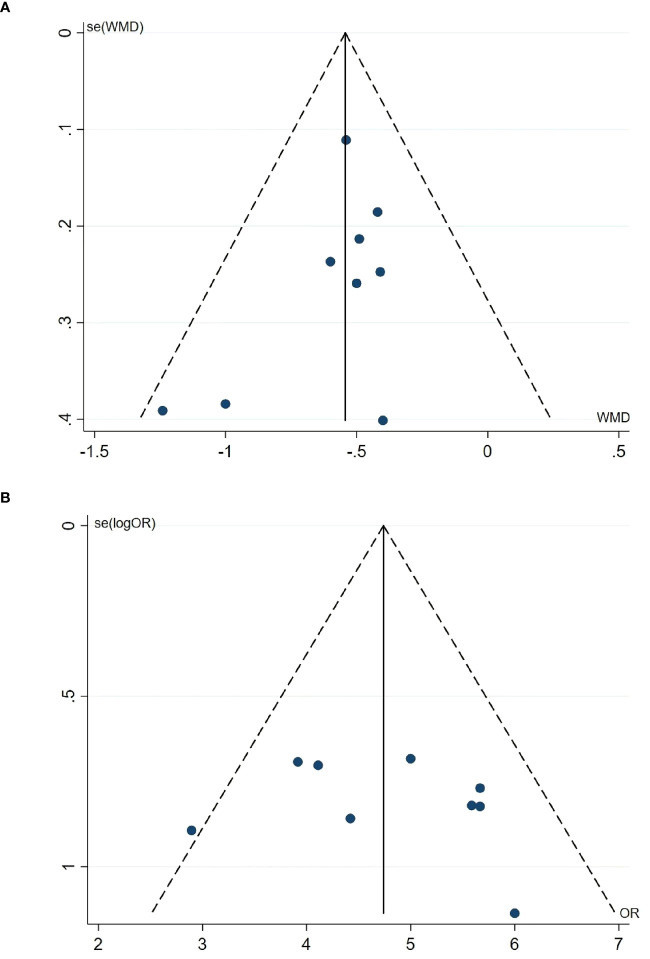
Funnel plot. **(A)** Funnel plot of the GET. **(B)** Funnel plot of the CE. GET, gastric emptying time; CE, clinical effect.

**Figure 6:**
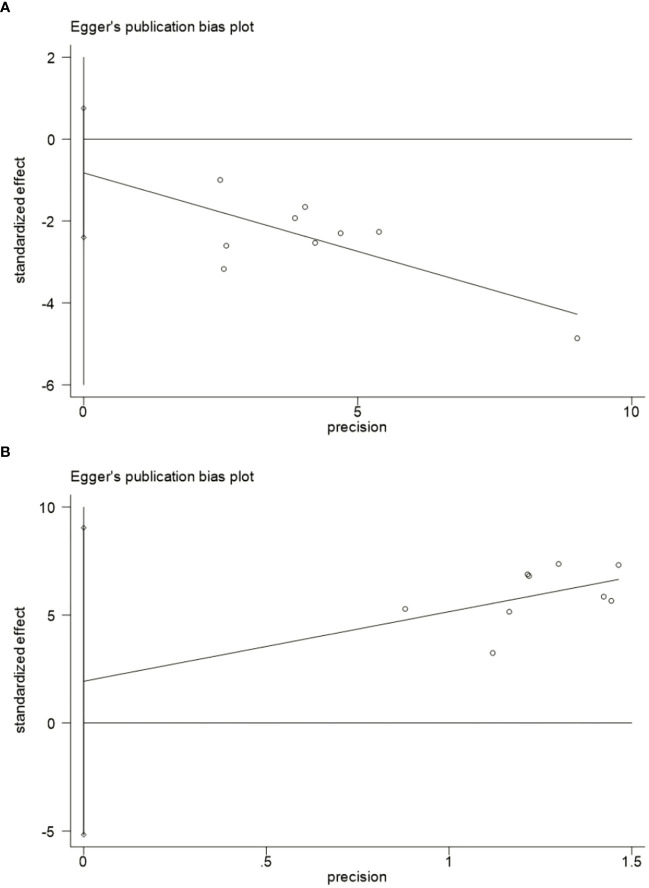
Egger’s publication bias plot.**(A)** Egger's publication bias plot for the GET. **(B)** Egger's publication bias plot for the CE. GET, gastric emptying time; CE, clinical effect.

Nine studies were included in the meta-analysis of GET and CE, so we evaluated their publication bias. The funnel plot (**Figure 5**) shows an approximate symmetry between them. Egger’s test (**Figure 6**) also indicated that no significant publication bias (GET: P= 0.256; CE: P= 0.540).

### 3.8 Quality of evidence

**Figure 7:**
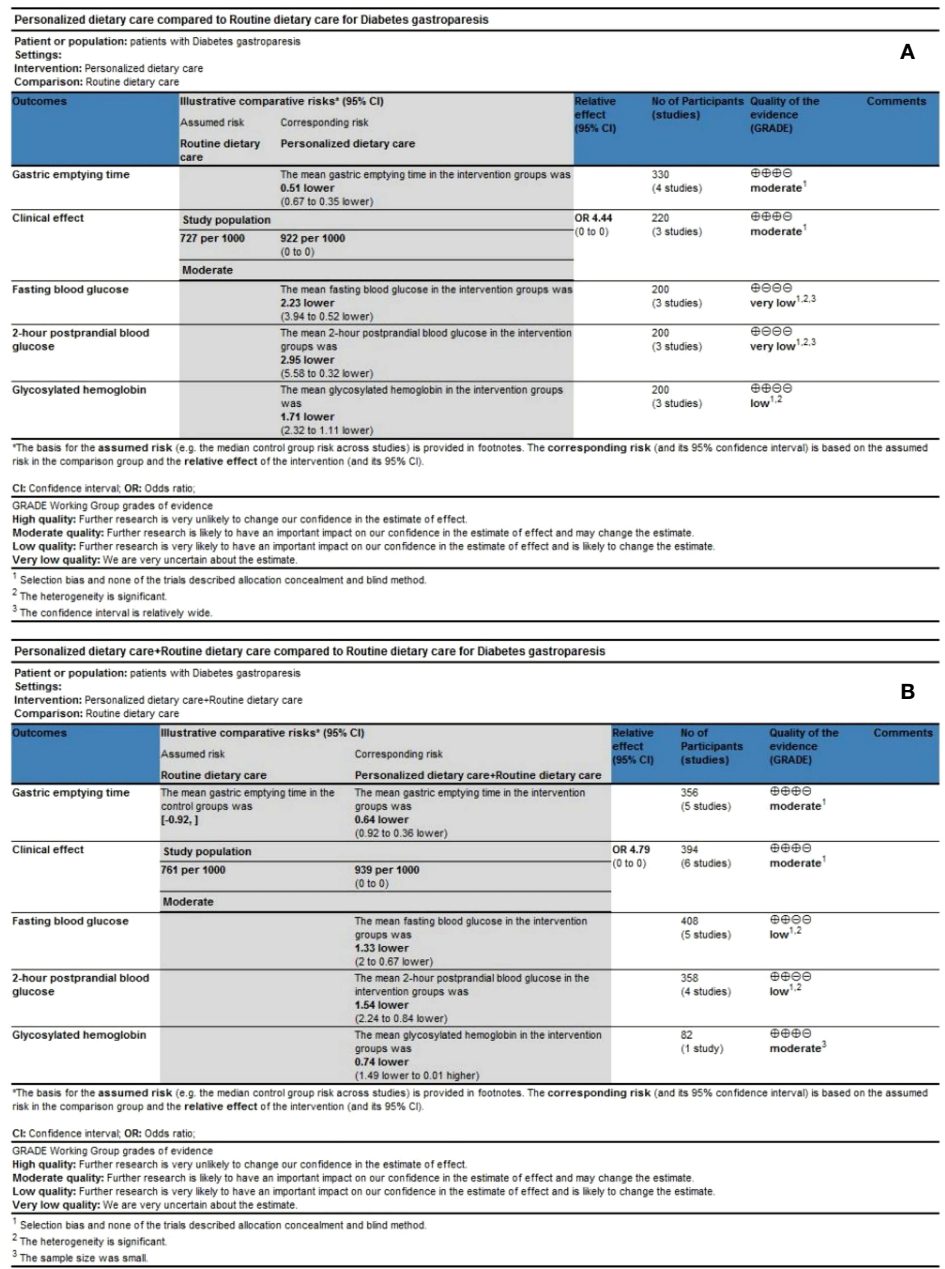
Quality of evidence. **(A)** GRADE evidence profiles for PDC versus RDC. **(B)** GRADE evidence profiles for PDC+RDC versus RDC. PDC, personalized dietary care; RDC, routine dietary care.

We used the Cochrane Collaboration Network GRADE approach to evaluate the quality of the meta-analysis results, including five results in the PDC group (**Figure 7A**) and five outcomes in the PDC+RDC group (**Figure 7B**). Evidence based on GRADE ratings showed that the quality of evidence for most outcomes was low or moderate, mainly associated with the risk of bias, high heterogeneity, and small sample size.

## 4 Discussion

It seems to be the first systematic review and meta-analysis to evaluate dietary therapy for DGP. This meta-analysis evaluated 15 RCTs of dietary interventions for DGP, including 1106 participants. Our analysis results showed that compared with RDC, PDC and PDC+RDC could shorten GET and promote gastric emptying better. Moreover, the CE of PDC and PDC+RDC group was better than that of RDC group. In addition, both PDC and PDC+RDC reduced FBG, 2hPBG and HbA1c levels in patients with DGP and improved blood glucose control. The stability of the overall results was also supported by sensitivity analysis and publication bias. However, in the three secondary outcomes of FBG, 2hPBG, and HbA1c, both the PDC group and the PDC+RDC group showed high heterogeneity. Sensitivity analysis seems to have identified the source of heterogeneity. In the PDC group, after excluding the study of Li W (28), the heterogeneity of FBG, 2hPBG, and HbA1c related research results was significantly reduced (I^2 ^= 0%), which may indicate the risk of bias and methodological quality issues in this study. However, in the PDC+RDC group, it was found that after excluding the study by Yu CJ et al. (36), the heterogeneity of the 2hPBG variable was significantly reduced (I^2 ^= 0%), which may be related to the risk of bias. Therefore, the results of the meta-analysis of FBG, 2hPBG, and HbA1c in the PDC group and the meta-analysis of 2hPBG in the PDC+RDC group should be treated with caution.

Although the quality of evidence is low to moderate, the findings suggest that personalized dietary interventions such as promoting a low-fiber diet, advocating the intake of liquid or semi-liquid foods, avoiding non-digestible foods such as fried and high-fat foods, following the principle of “a little each time but many times”, and adjusting the meal time and insulin injection time based on the patient’s postprandial blood glucose changes and gastric emptying. It can significantly shorten the GET of DGP patients, improve CE, reduce FBG, 2hPBG, and HbA1c levels.

DGP is an autonomic neuropathy caused by long-term poor blood sugar control (38). The independent risk factors for DGP include long-term hyperglycemia and high glycated hemoglobin (39, 40). There is a bidirectional relationship between blood glucose control and gastric emptying in DGP patients, that is, hyperglycemia-induced antral motility inhibition can delay gastric emptying, and delayed gastric emptying can also affect blood glucose control (41). Therefore, optimal control of blood glucose in patients with DGP may reduce the risk of future exacerbation of gastroparesis (1). The goal of blood glucose control should be controlled below 180 mg/dL to avoid inhibiting gastric myoelectric control and movement (38, 42).

Sympathetic and parasympathetic nervous system dysfunction, hyperglycemia and oxidative stress are all related to gastrointestinal motility dysfunction in diabetes (43, 44). Impaired gastric regulation in patient with diabetes is associated with decreased nitric oxide inhibitory innervation (45). Nitric oxide can affect pyloric dilation, and the loss of neuronal nitric oxide synthase may impair pyloric dilation and lead to pyloric spasm in patients with DGP (46, 47). This dysfunction (pyloric spasm) may be caused by vagus neuropathy and can hinder gastric emptying in DGP patients (48). Diet may have an impact on the regulation of the sympathetic nervous system, the release of nitric oxide, and vasodilation (49). A high-fat and carbohydrate diet can lead to activation of the sympathetic nervous system or regression of the parasympathetic nervous system, affecting the sympathetic nervous regulation of the heart (50). Research has shown that dysfunction of the cardiovascular vagus nerve can lead to delayed gastric emptying (51, 52).

Diet management is the first step in the treatment of gastroparesis patients (53, 54). Dietary interventions (including changes in dietary composition, size, and frequency of consumption, etc.) and stable blood sugar control are the fundamental management methods for DGP (7, 55). Dietary interventions can improve gastroparesis symptoms by regulating blood sugar control and promoting gastric emptying in DGP patients (56). Studies have shown that high-fat and high-fiber foods can delay gastric emptying, so patients with DGP should consume foods low-fat and low-fiber foods to compensate for the damage of gastric emptying (54, 57, 58). In addition, the diet of patients with DGP should be dominated by liquid or semi-liquid foods, as liquids are better tolerated than solid foods and are easier to empty (56, 59). Meanwhile, patients with DGP should eat small, frequent meals to maintain energy intake (54).

The PDC model of this review is different from the previous diet model of evaluating a single or several foods, because it systematically combines the power of multidisciplinary teams such as gastroenterologists, endocrinologists, nutritionists, psychologists, etc., which can more holistically and comprehensively evaluate the role of diet in DGP treatment. This review also has the following limitations. One is that the number of original studies related to dietary therapy for DGP is relatively small. Secondly, although the included studies have a consensus on PDC programs, there are also certain differences. However, these differences do not interfere with the positive effects of PDC in treating patients with DGP. Thirdly, the quality of evidence from the included RCTs was low to moderate. Fourthly, the included subjects are all from China. Considering the influence of dietary culture, religious customs and geographical environment, the results of this review should be cautiously applied to other countries and regions. Finally, due to significant differences in the treatment duration included in the study, this study did not discuss the length of intervention and the timepoint of evaluating intervention effectiveness.

Overall, the evidence from this systematic review and meta-analysis suggests that patients with DGP benefit from dietary interventions (whether PDC alone or PDC+RDC), which could shorten their GET, reduce their FBG, 2hPBG, and HbA1c levels, enhance their gastric emptying ability, and stabilize their blood sugar control. The most important measures in the dietary interventions program include the adjustment of the dietary composition of patients with DGP (advocating a low-fiber and low-fat diet), adjusting the dietary form (focusing on semi-liquid or liquid foods), and adjusting the number of meals (following the principle of “a little each time but many times”). In addition, other measures in the dietary interventions program are also an indispensable part.

## 5 Conclusion

The research results evaluated in this system review support the therapeutic potential of diet in the treatment of DGP. Both PDC and PDC+RDC effective for the treatment of DGP, as can promote gastric emptying by shortening GET, and also reducing FBG, 2hPBG, and HbA1c levels in patients with DGP to stabilize blood sugar control. However, the number of included clinical trials are relatively few and the methodological quality is average, and well-designed, larger sample RCTs are needed to further corroborate our findings and determine whether diet has a place in DGP treatment guidelines.

## Data availability statement

The original contributions presented in the study are included in the article/supplementary material. Further inquiries can be directed to the corresponding authors.

## Author contributions

DL: Conceptualization, Data curation, Formal analysis, Methodology, Software, Writing – original draft. HW: Conceptualization, Data curation, Formal analysis, Software, Writing – original draft. YO: Conceptualization, Formal analysis, Software, Writing – original draft. LL: Conceptualization, Data curation, Formal analysis, Writing – original draft. QZ: Data curation, Formal analysis, Writing – original draft. JY: Data curation, Writing – original draft. DP: Conceptualization, Methodology, Project administration, Writing – review & editing. SP: Conceptualization, Funding acquisition, Methodology, Project administration, Writing – review & editing.
